# Measurement of Serum Low Density Lipoprotein Cholesterol and Triglyceride-Rich Remnant Cholesterol as Independent Predictors of Atherosclerotic Cardiovascular Disease: Possibilities and Limitations

**DOI:** 10.3390/nu15092202

**Published:** 2023-05-05

**Authors:** Dieter Lütjohann, Hans-Ulrich Klör, Frans Stellaard

**Affiliations:** 1 Institute of Clinical Chemistry and Clinical Pharmacology, University Hospital Bonn, 53127 Bonn, Germany; 2 Department of Internal Medicine III, University of Gießen, 35392 Gießen, Germany

**Keywords:** chylomicrons, remnants, cholesterol, Friedewald equation, Martin–Hopkins equation, Sampson equation

## Abstract

The serum low density lipoprotein cholesterol (LDL-C) concentration is the dominant clinical parameter to judge a patient’s risk of developing cardiovascular disease (CVD). Recent evidence supports the theory that cholesterol in serum triglyceride-rich lipoproteins (TRLs) contributes significantly to the atherogenic risk, independent of LDL-C. Therefore, combined analysis of both targets and adequate treatment may improve prevention of CVD. The validity of TRL-C calculation is solely dependent on the accuracy of the LDL-C measurement. Direct measurement of serum LDL- C is more accurate than established estimation procedures based upon Friedewald, Martin–Hopkins, or Sampson equations. TRL-C can be easily calculated as total C minus high density lipoprotein C (HDL-C) minus LDL-C. Enhanced serum LDL-C or TRL-C concentrations require different therapeutic approaches to lower the atherogenic lipoprotein C. This review describes the different atherogenic lipoproteins and their possible analytical properties and limitations.

## 1. Introduction

**Figure 1:**
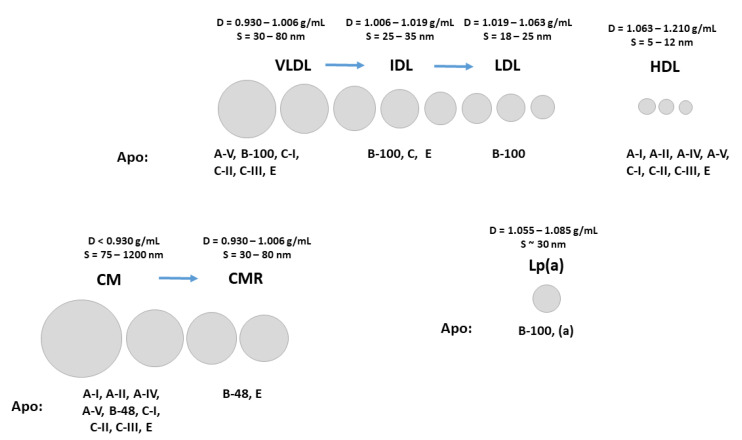
Lipoprotein distribution originating from the liver, i.e., very low density lipoprotein (VLDL), intermediate density lipoprotein (IDL), low density lipoprotein (LDL), lipoprotein (a) [Lp(a)], high density lipoprotein (HDL), and the intestine, i.e., chylomicron(CM) and CM remnants (R). The characteristics related to density (D; g/mL), size (S; nm) and lipoprotein composition are presented. Apolipoprotein (Apo) [15].

Increased serum total cholesterol (TC) is associated with an increased risk of developing atherosclerotic cardiovascular disease (ASCVD) [1,2,3]. High serum low density lipoprotein C (LDL-C) is generally considered as the predominant cause of ASCVD progression [4,5,6]. For decades, serum LDL-C has been the main target to be lowered with statins, either alone or in combination with ezetimibe [7]. Recently, bempedoic acid has been introduced as a possible replacement for statins when these cannot be tolerated by the patient [8]. In a considerable number of patients, LDL-C lowering targets are not reached [9,10,11]. Additional LDL-C lowering therapies have been developed, such as inhibition of proprotein convertase subtilisin/kexin type 9 (PCSK9), which intercellularly degrades the LDL-receptor (LDLR) [12,13]. LDLR is the carrier protein that enables LDL to enter the cell. LDL is formed by very low density lipoprotein (VLDL) through intermediate density lipoprotein (IDL) after progressive removal of triglyceride (TG) by lipoprotein lipase (LPL) and hepatic lipase (HL) [14] (Figure 1). VLDL, IDL, and LDL differ in particle size and density [15].

**Figure 2:**
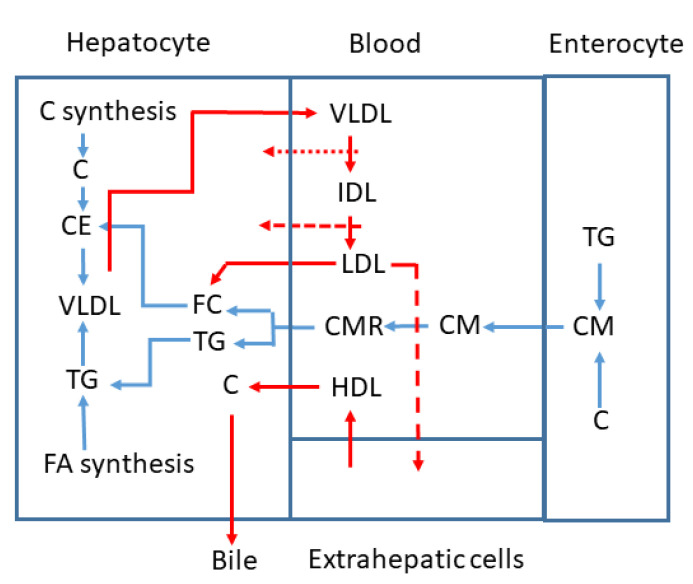
Formation of very low density lipoproteins (VLDL) and chylomicron (CM) lipid cores, secretion of VLDL and CM particles into blood and the blood metabolism of lipoproteins. Cholesterol, C; cholesterol ester, CE; triglyceride (TG); fatty acid (FA); intermediate density lipoproteins (IDL); chylomicron remnants (CMR); high density lipoproteins (HDL).

It is important to realize that VLDL, IDL, and LDL are density classes only composed of differently sized particles. Since VLDL secretion and LPL activity may vary over time, a large variety of lipoprotein particles with a range of TG and C contents is simultaneously present in serum [13,16,17,18]. These particles, which are larger and more TG-rich than LDL, are called TG-rich lipoproteins (TRLs). Additionally, highly TG-rich chylomicrons (CM) are continuously released from the intestine two and four hours after a fat-rich meal [19,20,21,22]. CMs are gradually converted to chylomicron remnants (CMR) via loss of TG by LPL. The sum of C in VLDL derived TRLs and CMs plus CMRs may be called total TRL-C. Recently, the measurement of remnant cholesterol (remnant C) has attracted considerable attention and its abundance has been proven to be associated with the development of various types of atherosclerotic events independent of LDL-C [23]. However, the definition of remnant-C and the determination of serum remnant-C concentration are subjects of discussion. According to the generally applied calculation, remnant C equals TRL-C. TRLs contain lipoprotein particles in between VLDL and LDL including IDL in combination with CMR [24]. All of these remnants and LDL are supposedly atherogenic. Graduation in atherogenicity cannot be clearly defined, despite the fact that small, highly dense LDL particles (“small-dense LDL”) are more atherogenic than larger ones [25]. In addition, while no clear general description of atherogenic lipoproteins can be provided, the presence of apolipoprotein B (ApoB) as carrier protein is at least characteristic. ApoB is represented as ApoB100 in VLDL-derived particles and ApoB48 for chylomicron-derived particles. This separates atherogenic particles from high density lipoprotein (HDL) which carries apolipoprotein A1 (ApoA1) as the unifying apolipoprotein. Additionally, the cholesterol ester (CE) content adds further to the atherogenicity [26,27,28]. Different risk indicators have been introduced to predict atherosclerotic risk. Apart from clinical indicators, such as obesity, smoking and/or diabetes, these include the serum concentrations of LDL-C, VLDL-remnant C (TRL-C), non-HDL-C and ApoB. ApoB has been introduced as a risk indicator based on the knowledge that CMs, CMRs, TRLs, and LDLs contain one ApoB molecule per particle and that the number of particles may be more conclusive than the concentration of lipoprotein C [29,30]. To date, clinicians tend to rely on LDL-C as the best marker for pro-atherogenic lipoproteins and HDL-C as the marker for anti-atherogenic lipoproteins [31,32,33]. It should be realized that HDL particles exchange CE with ApoB-containing particles in exchange for TG [34] mediated by cholesterol ester transfer protein (CETP). Reduction of CETP activity is considered a potential target for increased reversed cholesterol transport [35]. Thus, depending on CETP activity the TG and CE proportions in HDL and ApoB lipoproteins may vary. HDL mainly delivers phospholipids and CE to the liver, whereas ApoB remnants and LDL are taken up to some extent by extrahepatic tissues, but predominantly by the liver via the LDL-receptor (LDLR) and the LDL-receptor related protein (LRP) [36]. One particular lipoprotein is the lipoprotein (a) (Lp(a)). It represents the densest ApoB-100-containing particle with a density higher than LDL. The measured LDL-C contains C originating from Lp(a). The lipoprotein lipid metabolism is presented in Figure 2.

**Figure 3:**
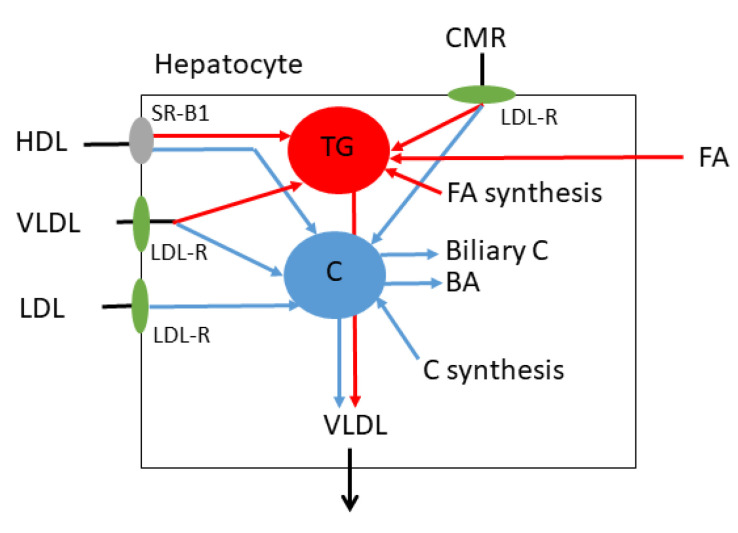
Hepatic TG and C metabolism. High density lipoprotein, HDL; very low density lipoprotein, VLDL; low density lipoprotein, LDL; scavenger receptor, class B type 1, SR-B1; LDL receptor, LDL-R; cholesterol, C; bile acids, BA; chylomicron remnants, CMR; fatty acid, FA.

VLDL is formed in the liver and transports TG and CE into the blood. It is gradually converted into LDL via intermediate formation of IDL. VLDL remnants and IDL may partly return to the liver before being converted to LDL. LDL is extracted into extrahepatic cells, but predominantly into the liver. CMs are produced in the enterocyte, transporting TG and CE from absorbed fatty acids from the diet and FC from the diet and from bile. They are converted to CMRs by the action of LPL and then delivered to the liver. The TG content of VLDL is provided by TG derived from CMR, fatty acids (FA) synthesized in the liver and FA taken up from blood (Figure 2 and Figure 3). The hepatic C pool is composed of C derived from extracted HDL, LDL, VLDL remnants, IDL and CMR as well as from synthesized C. Hepatic TG is secreted into the blood in VLDL particles. Hepatic C is secreted in VLDL as CE, secreted into bile as FC, and as bile acids. The distribution of C divided over these three fluxes is largely unknown. HDL-C appears to be dominantly secreted into bile [37,38] and converted to bile acids [39]. The flux distribution may be dependent on the hepatic C concentration. In this review we critically evaluate the proposed predictive markers according to the characteristics of various lipoproteins, their metabolism, and their analysis or calculation procedures.

## 2. Atherogenic Lipoprotein C Concentrations as Indicators of Enhanced Risk for Atherosclerosis Development

The pro-atherogenic character of lipoproteins is not yet fully understood. In principle, HDL particles are considered anti-atherogenic. Accepted atherogenic characteristics are the presence of ApoBs (ApoB100 and ApoB48), reduced size and increased density beyond undefined limits, and a high load of CE. To actually cause atherosclerosis, the turnover of lipoproteins must be delayed. This increases the exposure of the arterial wall to the toxic lipoproteins, thus enhancing the atherosclerotic process. A delay of turnover may be caused by reduced activity of LPL, HL, and/or LDLR. During the day, the presence of intestinal-derived CMs and CMRs is maximized with fat-rich meals. Therefore, lipoprotein analysis in fasting blood will not represent the daily exposure to atherogenic lipoproteins. The time to transport CMs, convert CM to CMR, and transport CMR to the liver affect the time-dependent contribution of CM-derived lipoproteins in the postprandial phase. Thus, the residence time of TRLs in plasma determines an individual’s atherosclerotic risk. Patients with obesity, diabetes mellitus, kidney disease, and/or familial history of cardiovascular disease have an increased risk of developing ASCVD. This risk must be conveyed to the patient and followed by treatments for risk reduction. After cardiovascular events have been treated, the residual risk for repeated events must be considered. Continuous treatment is required to reduce any residual risk. The degree of atherogenicity is associated with the level of circulating C-rich lipoproteins (LDLs, remnants, Lp(a)). Initially, serum TC was used as a predictive marker and serum C lowering therapies were developed as the treatment of choice. Thereafter, the target became the lowering of serum LDL-C concentration. In the last decade, initiatives were undertaken to extend the focus to the other potentially atherogenic lipoprotein C, by applying non-HDL-C as the predictive marker [40]. Non-HDL-C is calculated as TC minus HDL-C and contains C present in all potentially atherogenic lipoproteins, including LDLs, remnants and Lp(a). Additional evidence identified the C content of the TRLs, i.e., all ApoB containing lipoproteins, excluding LDL-C, were an additional risk marker in patients with normal LDL-C levels or those with a sufficiently reduced LDL-C level following C-lowering treatment [41]. A third suggestion has been to measure total ApoB, i.e., ApoB-100 plus ApoB-48 as a risk marker [42,43]. Lp (a) is an independent risk marker in specific patients and should always be measured during the original diagnosis. Next, the validity of various markers is discussed.

## 3. LDL-C

For many years, a high serum LDL-C concentration has been considered the central factor associated with an increased risk for development of atherosclerosis and cardiovascular events [1,3,5]. The definition of LDL-C is the lipoprotein consisting of Apo B-100 as the associated apolipoprotein (20% of total content), with a density of 1.019 to 1.063 g/mL and a diameter of 20 to 25 nm. The lipid core contains 12% TG and 59% cholesteryl esters. Small LDL particles are more atherogenic than larger ones [44]. The LDL-C concentration is determined in every hospital all over the world. For the most accurate analysis of LDL-C, serum must be treated with ultracentrifugation [45,46] in order to isolate the LDL density fraction for C analysis. This is the official reference method, which is considered optimally selective. A second approach to separate lipoproteins is via electrophoresis [47] and a third approach is via nuclear magnetic resonance (NMR) [48]. In addition, liquid mass spectrometry methods are now being developed [49]. However, for routine clinical laboratories, these techniques are time consuming, laborious, and expensive. In 1972, Friedewald established a simple estimation procedure [50]. In fasting serum, total C is predominantly composed of HDL-C, LDL-C, and VLDL-C (plus IDL-C). From measurement of VLDL-C and VLDL-TG after isolation with ultracentrifugation, Friedewald found that the mean TG/C ratio in fasting serum was 5.0 and considered this number to reflect the ratio in the healthy population. Thus, he expressed the LDL-C calculation as: LDL-C = TC minus HDL-C minus TG/5. Obviously, the Friedewald formula means that the calculated LDL-C strongly depends on the TG concentration. TG is bound to the above-mentioned lipoproteins and is not limited to VLDL. VLDL contains about 55% TG. CMs carry 88% TG, which decreases during conversion to CMR. The TG content of lipoproteins is dependent on VLDL and chylomicron production and release as well as LPL activity. Furthermore, CE is transferred from HDL into LDL in exchange for TG. From VLDL, CM, and CMR, low LPL activity leads to high TRLs. It has been observed that the Friedewald equation starts to lose accuracy when the LDL-C concentration is low (<1.8 mmol/L, <70 mg/dL) and the TG concentration is high (>1.69 mmol/L, >150 mg/dL). Generally, it is acceptable to use the Friedewald equation when LDL-C > 1.4 mmol/L (>54 mg/dL) and the TG concentration < 4.5 mmol/L (<400 mg/dL) and Lp(a) is within the reference range [51]. This makes the equation unsuitable in patients with hypertriglyceridemia and mixed hyperlipidemia and increased Lp(a) levels. Furthermore, recent literature advocates lipoprotein profiling to be performed in the postprandial phase, when TG is at higher levels and CMs and CMRs are present at variable higher concentrations [52,53,54,55]. Recent evidence has been provided indicating that production and release of chylomicron particles are slow processes. Fat is temporarily stored as liquid droplets in the intestinal cells [56]. The supply of chylomicrons and as a consequence chylomicron remnants are spread out over time. This way, the body is protected against an excessive load of fat after meal consumption. Depending on the dietary fat intake, the time point of the last meal, and the delay of CM secretion, the presence of CMs and CMRs in fasting serum may become relevant. Approaches have been made to improve the weakness of the Friedewald equation. The most accepted improvements are the approaches of Martin [57] and Sampson [58]. They correct the LDL-C value according to the combination of TG and HDL-C in the sample. Both approaches extend the range of TG concentrations at least up to 9 mmol/L (800 mg/dL). The Martin–Hopkins approach also provides more accuracy at low LDL-C concentrations. However, direct measurement of LDL-C is highly recommended. Many commercially available direct homogeneous LDL-C assays are on the market [59], enabling a rapid and selective analysis. These assays are based on the fact that they exclude HDL, VLDL, and CMs from the C measurement. However, CM- and VLDL remnants with reduced TG content—if present in fasting serum—may potentially interfere with the measurement. Another potentially interfering factor is LP(a). LP(a) equals LDL in size and density and may be included in the measurement of LDL-C if present.

## 4. TG Rich Lipoprotein C (TRL-C) or Remnant C

Almost three decades ago it was indicated that TG enriched lipoproteins (TRL-C) in serum correlate with the severity of coronary artery disease [60]. Serum TRL-C, also called remnant C, received much attention as an atherogenic component associated with cardiovascular events and independent of serum LDL-C. In 2022, over 2000 hits were obtained when searching the Pubmed data base for “remnant cholesterol”. A small extract is shown here [61,62,63]. It has frequently been proposed that “remnant C” should be determined in the individual patient at risk and that remnant-C lowering therapies need to be established. Interestingly, according to the calculation procedure, remnant-C equals TRL-C in fasting serum. Therefore, we will continue using the term TRL. Elevated serum TRL concentrations may be caused by various factors, such as excess dietary TG intake, high secretion rates of CMs, high hepatic VLDL secretion, and most importantly, by reduced efficiency of LPL [14,64,65]. A high serum TRL concentration is most likely the result of the combination of enhanced secretion and reduced lipolysis. This will initially result in relatively large TG rich particles that may be less atherogenic. In the extreme situation of genetically caused inhibition of LPL, hyperlipidemic pancreatitis is more common than ASVD [66,67]. As indicated before, TRL-C is calculated as TC minus HDL-C minus LDL- C. When the Friedewald formula is used for the LDL-C calculation, the inaccuracy in the determination of LDL-C affects the TRL-C calculation. As a matter of fact, the equation can then be rewritten as TRL-C = TG/5. In a healthy situation, the TRL-C concentration calculated via the Friedewald equation is on average about 10% of the LDL-C concentration. The LDL-C concentration calculated by the Friedewald equation tends to underestimate LDL-C by about 10% when compared to LDL-C measurement after LDL isolation using ultracentrifugation [68]. Correcting LDL-C for a potential 10% underestimation leads to about 50% reduction in TRL-C. This suggests using direct measurement of LDL- C to calculate a reliable TRL-C concentration. Recently Varbo et al. [69] described an alternative technique to measure TRL-C independent of LDL-C and HDL-C. Using a commercial assay (Denka, TRL-C, Denka Company Limited, Tokyo, Japan), LDL and HDL are degraded and removed. Thereafter C is measured. It was found that directly measured TRL-C identified 5% more patients with increased risk of cardiovascular disease than calculated TRL-C applying the Martin–Hopkins equation. The question arises as to how the patients involved should be treated. Probably, their clinical and nutritional status must be closely studied. An obese patient with a high fat intake may be successfully treated by reduction of dietary fat intake. This may be achieved with a low fat, fiber rich diet, eventually combined with orlistat, which binds to pancreatic lipase reducing fat digestion and promotes weight loss [70,71,72]. A patient with high sugar intake may limit sugar intake and thereby potential endogenous fat synthesis. New therapies are under development such as pemafibrate [73] and the omega-3 fatty acid icosapent ethyl [74]. Decreased serum TG was observed under statin treatment and more pronounced under combination of statin with ezetimibe [75,76]. The mechanism for this serum TG reduction during LDL-C reduction therapy is unclear.

## 5. Non HDL-C and ApoB

Interestingly, discussion has focused serum LDL-C and TRL-C as determinants of increased cardiovascular risk. Apparently, a subgroup of patients develops cardiovascular disease despite a normal LDL-C concentration [1]. Other research groups promote non- HDL-C as the ultimate marker of atherogenic cardiovascular disease risk. Non-HDL-C is a calculated parameter obtained as non-HDL-C = TC − HDL-C. TC as well as HDL-C are measured directly with generally accepted methods. The difference is well defined and without discussion. It reflects LDL-C plus TRL-C. Thus, non-HDL-C contains all atherogenic components. However, large and potentially less atherogenic TRL components may be included, particularly when LPL activity is low. This may decrease the prognostic efficiency. Furthermore, at TG > 400 mg/dL, HDL-C measurement is inaccurate since TRLs are not sufficiently precipitated and thus TRL-C is partly included in the HDL-C value. Normally, LDL-C comprises the majority of non-HDL-C. However, non-HDL-C is considered a better predictor for a residual risk for cardiovascular disease than LDL-C [40]. It is also known that an undefined subgroup of ApoB-containing lipoproteins expresses the highest atherogenic action and it has been established that small dense LDL particles are more atherogenic than larger ones. Thus, the number of lipoprotein particles reflects the atherogenicity better than the lipoprotein concentration. Since each ApoB-containing lipoprotein carries only one ApoB molecule, it has been proposed to determine the total ApoB concentration as a measure of atherogenicity [43,77], also under statin treatment [78]. This uncouples atherogenicity from C. Apo-B and Apo-AI can be assayed using commercial test kits based on automated immunoturbidimetric methods (Randox, Crumlin, United Kingdom). First, Apo-B-containing particles are precipitated from serum by phosphotungstic acid–MgCl_2_. ApoA1 is measured in this fraction while ApoB in the residual fraction. For optimal differentiation, the separation of ApoB100 from ApoB48 may be considered in distinguishing between liver-derived and gut-derived ApoB containing TRL particles.

## 6. Personalized Diagnostics and Therapy

This review highlights a discrepancy between available research findings and daily clinical routine. It may take some time before the measurement of TRL-C and ApoB concentrations in serum become routine analysis in the clinical laboratory. Routine daily measurements include TC, TG, HDL-C, and LDL-C. Measurement of LDL-C via direct methodology is slowly being introduced and must be further standardized in all clinical laboratories. While LDL-C may remain a leading predictive parameter, the additionally acquired data for TRL-C should also be considered. Table 1 outlines a diagnostic and personalized treatment strategy.

It must be realized that Lp(a) is included in LDL. Therefore, this has to be measured in each patient at least once in a lifetime. LDL-C lowering should consist of combined statin or bempedoic acid and ezetimibe treatment in order to obtain the maximal response. When LDL-C lowering is insufficient, PCSK9 inhibition should be added to the combination treatment.

## 7. Limitations

The proposed extended diagnosis procedure of the atherogenic lipoprotein components depends to a large extend on the quality of the LDL-C measurement. Isolation of LDL using ultracentrifugation followed by C measurement will ensure ultimate quality. However, this technique is too time consuming and complex to be incorporated into daily clinical routine. Homogeneous direct assays are now available to isolate LDL by chemical means [59,79]. However, these various commercial assays may produce different results. In addition, the validity of the assay appears high in healthy subjects and lower in patients with cardiovascular disease [80]. Furthermore, the analytical result may differ when measured in fresh serum or frozen serum. Therefore, the most reliable assay must be chosen and applied under controlled conditions. Any reasoning for applying the measurement must be well defined. When the risk of development of cardiovascular disease needs to be determined by measuring atherogenic lipoprotein C in documented patients, LDL-C measurement must be performed exactly under cardiovascular conditions. A concentration above the cut off level of the normal range is the criterion for treatment. It will suffice to apply the same assay and quality control criteria continuously. The patient should be followed over time during treatment.

## 8. Summary of Results

VLDL and CM-derived remnants including CMR, IDL, and LDL in serum are considered atherogenic. Their C concentrations in serum are documented as predictive atherogenic indicators of cardiovascular disease risk. VLDL, IDL, and LDL are the dominant lipoproteins in fasting serum. CMRs are added to postprandial serum in amounts depending on the dietary fat intake. Serum LDL-C is used as the gold standard for risk prediction and treatment is focused on lowering serum LDL-C. VLDL-C, IDL-C, and CMR-C are called TRLs. Atherosclerosis may develop in patients with low LDL-C and high TRL-C concentrations. The accuracy of LDL-C and TRL-C determinations is procedure-dependent, i.e., on direct measurement or estimation procedures. It is unknown whether all TRLs are equally atherogenic. Non-HDL-C combines TRL-C and LDL-C and thus all potentially atherogenic lipoprotein species. Non-HDL-C may be considered the best and simplest marker of lipoprotein atherogenicity. At higher serum TG concentrations (>300 mg/dL) HDL-C also includes TRL-C because those particles are not completely precipitated by the HDL-determination method. LDL-C and TRL-C concentrations in fasting serum do not usually reflect the daily exposure to atherogenic lipoproteins, which is highest in the postprandial phases. Potentially atherogenic lipoproteins all contain ApoB. The serum ApoB concentration reflects the number of atherogenic particles and thus the cardiovascular risk. Patients with elevated serum TRL-C concentrations may be detected when serum LDL-C is measured directly with sufficient accuracy. Homogeneous, direct assays are available allowing rapid analysis in a clinical routine setting. However, selection of the preferred assay must be performed carefully.

## 9. Conclusions

Epidemiologic and genetic studies have established TRL and their remnants as important contributors to ASCVD. Combinations of LDL-C, non-HDL-C, TRL-C, and ApoB concentrations must be evaluated as the utmost predictive risk marker for development of cardiovascular disease and are recommended in the current guidelines. For clinical routine, direct measurements of TGs, TC, HDL-C, and LDL-C allow semi accurate calculation of TRL-C and non HDL-C. Patients with elevated LDL-C may be treated with conventional C lowering therapies. Patients with elevated TRL-C should be detected and treated specifically. The first step of treatment is the implementation of lifestyle interventions. Second, LDL-C lowering with statins or bempedoic acid—in case of statin intolerance—with or without ezetimibe are recommended to reduce vascular risk, independent of statin-associated lowering of TRL itself. Novel and emerging data, e.g., on omega-3 fatty acids (high-dose icosapent ethyl) and new generations of selective peroxisome proliferator-activated receptor (PPAR) modulator pemafibrate may identify patients who will benefit from TRL lowering.

## Figures and Tables

**Table 1:** A proposed scheme of diagnosing the cause of development of atherosclerosis via elevated serum LDL-C or serum TRL-C. Personalized treatment can be applied.

Total C	LDL-C	TRL-C	Therapy
Normal	Elevated	Low	LDL-C lowering
Normal	Low	Elevated	TG-lowering
Elevated	Elevated	Normal	LDL-C lowering
Elevated	Normal	Elevated	TG-lowering
Elevated	Elevated	Elevated	LDL-C and TG-lowering

C, cholesterol; LDL, low density lipoprotein; TRL, triglyceride-rich lipoproteins; TG, triglyceride.
